# A New Operating Chair

**Published:** 1849-07

**Authors:** 


*A New Operating Chair.*—Mr. Gilbert, a medical practitioner, has recently invented and *patented* in England, a chair with a fulcrum to be used in extracting teeth. It consists of a padded and easy seat, with arms, a movable back, which lets down to any inclination, is semi-circular for receiving the head of the patient. At the right hand side is screwed a strong circular steel bar; on this runs another at right angles, which is fixed by an adjusting screw at any required length, the cross bar at its termination holds a flat piece of steel, which is brought opposite to or within the mouth, and which is intended as the fulcrum for the forceps to rest upon in operating, when employed for the extraction of teeth in the lower jaw, the forceps are placed superiorly to this fulcrum. When used for the upper jaw the chair is let down in a horizontal position and the operator stands behind making the inferior surface of the bar serve as the fulcrum. The forceps having grasped the tooth, the operator (so says the patentee) with a single movement, perpendicularly, raises the tooth.

Mr. Gilbert states, "that he was led to the construction of his invention, which he has now fully matured, by a remark of John Hunter, to the end that the extraction of a tooth should, 'if possible,' be effected perpendicularly, or *in the direction of its axis.* (See 'Natural History of the Teeth,' p. 122.) Every surgeon and anatomist must needs admit, that the more extensively a *lateral* force is used, the greater is the extent of injury to the alveolar process, to say nothing of the increased pain and hemorrhage consequent upon the crashing the jaw and the structures by which it is surrounded, which is inevitable unless the fulcrum can be removed from these structures."

Without in the least detracting from the ingenuity displayed by Mr. Gilbert in the conception and construction of his chair, we are prepared to prove, that its practical uses to the dentist are extremely limited, even when applied to the lower jaw, and as to its employment in the upper, for *perpendicular extraction,* we believe every practical professional man will agree with us, it is utterly impossible to extract any one class of human teeth without using the alternate lateral motion, still much praise is due to Mr. G. for the ingenuity of purpose, still the same theory of perpendicular extraction has heretofore been attempted, and signally failed—in the key instrument, with circular bolster, invented by Mr. Inglis some fifteen years since. R.

